# Cytokine polymorphisms influence treatment outcomes in SLE patients treated with antimalarial drugs

**DOI:** 10.1186/ar1897

**Published:** 2006-02-13

**Authors:** Patricia López, Jesús Gómez, Lourdes Mozo, Carmen Gutiérrez, Ana Suárez

**Affiliations:** 1 Department of Functional Biology, Area of Immunology, University of Oviedo. Spain; 2 Department of Immunology, Hospital Universitario Central de Asturias, Oviedo. Spain

## Abstract

Antimalarial agents have been widely used as disease-modifying antirheumatic drugs in the treatment of systemic lupus erythematosus (SLE) and other rheumatological diseases, although their mechanism of action has not yet been fully defined. It is known, however, that effective response to treatment is variable among patients. Thus, the identification of genetic predictors of treatment response would provide valuable information for therapeutic intervention. The aim of the present study was to analyze the effect of antimalarial treatment on tumor necrosis factor (TNF)α serum levels and evaluate the possible influence of TNFα and IL-10 functional genetic polymorphisms on the response to antimalarial drugs. To this end, TNFα serum levels were quantified in 171 SLE patients and 215 healthy controls by ELISA techniques and polymorphisms at positions -1,082 and -308 of the IL-10 and TNFα gene promoterswere determined by PCR amplification followed by hybridization with fluorescent-labeled allele-specific probes in 192 SLE patients and 343 matched controls. Data were related to clinical features and treatment at the time of sampling and during the course of the disease. Results showed a significantly higher amount of serum TNFα in the entire SLE population compared with controls. However, TNFα serum levels correlated negatively with the use of antimalarial treatment during at least three months before sampling. Patients under single or combined treatment with these drugs had TNFα serum levels similar to healthy controls, whereas untreated patients and those under corticosteroid or immunosuppressive therapies had increased amounts of this cytokine. This suggests, however, that antimalarial-mediated inhibition of TNFα was only significant in patients who were genetically high TNFα or low IL-10 producers. In addition, evaluation of SLE patients administered antimalarial drugs for three or more years who did not require any other specific SLE treatment indicates that patients with the combined genotype low IL-10/high TNFα are the best responders to antimalarial therapy, developing mild disease with a good course under this treatment. In conclusion, we proposed that an antimalarial-mediated downregulation of TNFα levels in SLE patients is influenced by polymorphisms at IL-10 and TNFα promoters. Our results may thus find important clinical application through the identification of patients who are the most likely to benefit from antimalarial therapy.

## Introduction

Systemic lupus erythematosus (SLE) is a disorder of immune regulation resulting in chronic inflammation that affects many organs. Treatment of lupus disease must be determined individually, since different patients may have diverse and multiple symptoms with variable severity. Mild disease requires no or little therapy, usually nonsteroidal anti-inflammatory medications (NSAIDS). Articular and skin symptoms are frequently treated with antimalarial drugs, especially hydroxychloroquine, alone or with low dose corticosteroids when required, whereas severe lupus must be treated with corticosteroids and/or immunosuppressive drugs. However, it is assumed that responses to specific agents may be variable among SLE patients. Consequently, the identification of genetic predictors of treatment response would provide valuable clinical information, since these can be determined at the time of diagnosis, when therapeutic intervention has the potential to offer the greatest benefits.

Antimalarial drugs (hydroxychloroquine, chloroquine and quinacrine)have been used as disease-modifying antirheumatic agents in the treatment of several autoimmune diseases, usually associated with increased secretion of pro-inflammatory cytokines. However, despite an extensive clinical history of use in rheumatoid arthritis and SLE [1], their mechanisms of anti-inflammatory action have not yet been fully defined. Chloroquine is thought to concentrate in acidic subcellular compartments, such as endolysosomes, where it inhibits acidic proteases [2]. As lysosomal enzymes are involved in antigen processing and presentation, an antirheumatic effect might be mediated by a downregulation of the immune response against autoantigens. However, the effects of chloroquine and related drugs may extend beyond this. Interestingly, antimalarials have been shown to inhibit the release of the pro-inflammatory cytokines IL-1, IL-6 and tumor necrosis factor (TNF)α by monocytes activated with lipopolysaccharide (LPS) or CpG oligonucleotides [3-6] and it has been reported that chloroquine interferes with LPS-induced expression of the gene encoding TNFα in human blood monocytes by a nonlysosomotropic mechanism [7]. However, it is not yet known whether treatment of humans with antimalarial drugs is capable of reducing pro-inflammatory cytokines *in vivo*.

Considering the central role that IL-10 and TNFα cytokines play in the pathogenesis of SLE, it is possible that different cytokine production may not only affect the natural course of the disease, but also the response to therapy. Genetic polymorphisms at the promoter of the genes encoding IL-10 and TNFα have been associated with different constitutive and induced cytokine production. The genetic variant at position -308 (G/A) of the gene encoding TNFα was found to have functional effects on gene transcriptional activity, carriers of the uncommon TNF2 allele (-308A*) being considered as genetically high TNFα producers [8-10]. Similarly, IL-10 basal and induced production presented interindividual variations that were genetically regulated by three single nucleotide polymorphisms (SNPs) at positions -1,082(G/A), -819(C/T) and -592(C/A) of the IL-10 promoter. In Caucasian populations, only three haplotypes have been found (GCC, ACC and ATA), the individuals GCC/GCC being considered as genetically high IL-10 producers [10-12]. Several studies have analyzed the association of IL-10 or TNFα genetic variants with susceptibility to and outcome of SLE and other autoimmune diseases, showing variable results in most cases. However, it is known that the actions of cytokines may be profoundly conditioned by the presence of other cytokines, this being particularly true in the case of IL-10 and TNFα, which are mutually regulated and have complex and predominantly opposing roles in systemic inflammatory responses. In a previous study, we found that carriers of the combined genotype high TNFα/low IL-10 have the highest risk factor for developing SLE and producing antibodies to the SSa antigen [10], suggesting that interactions between IL-10 and TNFα cytokine genes may influence susceptibility to SLE, its phenotype and possibly the clinical response to disease modifying antirheumatic drugs. The aim of the present study was to detect the effect of SLE treatments on TNFα serum levels and to evaluate the possible influence of IL-10 and TNFα functional genetic polymorphisms on the response to antimalarial drugs.

## Materials and methods

### Patients

Approval for this study was obtained from the Regional Ethics Committee for Clinical Investigation. Patients included in the study (*n *= 192) were from the Asturian Register of SLE [13]; all of them were Caucasian in origin and fulfilled the American College of Rheumatology (ACR) criteria for SLE [14]. At the time of serum sampling for TNFα quantification, patients were asked precise questions regarding the treatment received during the past three months. All untreated or NSAID treated patients presented inactive SLE. In addition, for genotype associations, information on clinical manifestations (age at diagnosis, disease duration, malar rash, discoid or subacute cutaneous lesions, photosensitivity, oral ulcers, arthritis, serositis, renal, neurological or hematological disorder) and treatments followed during the course of the disease was obtained after a detailed review of clinical histories. Those patients receiving antimalarial agents for three or more years without requiring any other specific treatment were defined as good responders to antimalarial therapy. The demographic and clinical characteristics of the patients are shown in Table 1. Matched healthy controls (*n *= 343) were obtained from the Asturian Blood Transfusion Center. Consent was obtained from all individuals prior to participation in the study.

### TNFα quantification

Serum samples for TNFα quantification were collected from 171 SLE patients and 215 healthy controls. TNFα concentration was determined by an in-house ELISA test, as follows. Microtiter wells were coated overnight with affinity purified anti-human TNFα monoclonal antibody (R&D Systems, Abingdon, UK) and blocked with 1% casein in Tris Buffered Saline (TBS) for two hours at 37°C. Samples and TNFα standards (R&D) were diluted in blocking solution and incubated for 18 hours at 4°C. After washing with TBS/Tween 20 (0.05%), wells were incubated for two hours with biotinylated anti-human TNFα monoclonal antibody (R&D), washed, incubated for one hour with streptavidin-alkaline phosphatase conjugate and revealed using *p*-nitrophenyl phosphate as substrate. Absorbance was determined at a wavelength of 405 nm. Quantities of serum TNFα were calculated according to the standard curves. The assay has a detection limit of 7.5 pg/ml, a within-run imprecision (coefficient of variation) of <7%, and a between-run coefficient of variation of <10%.

### Promoter polymorphism genotyping

DNA was obtained from the peripheral blood cells of 192 SLE patients and 343 local Caucasian unrelated healthy blood donors by standard procedures. SNPs at positions -1,082 on the gene encoding IL-10 and -308 on the gene encoding TNFα were determined by analyzing the Tm of the probe/target duplex after PCR amplification and hybridization with fluorescent-labeled probes matched with one sequence variant (LighCycler, Roche Diagnostics, Mannheim, Germany), as was previously reported [10]. The primers used were: 5'-ATC CAA GAC AAC ACT ACT AAG GC and 5'-ATG GGG TGG AAG AAG TTG AA for -1,082 IL-10 and 5'-CCT GCA TCC TGT CTG GAA GTT A and 5'-CTG CAC CTT CTG TCT CGG TTT for -308 TNFα. The hybridization probes (designed by TIB MOLBIOL, Berlin, Germany) were: GGA TAG GAG GTC CCT TAC TTT CCT CTT ACC-F and LC Red 640-CCC TAC TTC CCC CTC CCA AA for -1,082 IL-10 and AAC CCC GTC CCC ATG CCC C-F and LC Red 640-CCA AAC CTA TTG CCT CCA TTT CTT TTG GGG AC for -308 TNFα.

### Statistical analysis

As serum TNFα levels were not distributed normally, nonparametric testing was used throughout (Mann-Whitney *U *test or Kruskal-Wallis test). Correlations between TNFα concentration and clinical parameters were performed using Spearman's rank correlation test. TNFα values were described by median and interquartile range. Univariate and multivariate analyses were performed by unconditional logistic regression to define the impact of specific single or combined functional genotypes on the response to antimalarial treatment, calculating odds ratios (ORs) and 95% confidence intervals (95% CI). Single-locus regression models were run to estimate the effects of IL-10 and TNFα cytokine polymorphisms separately, comparing the high producer genotypes with the most common low producers. A combined two-loci model was developed, including both cytokine polymorphisms to estimate individual effects of each combined genotype, using the common low/low producer genotype as referent. Covariates for the multivariate analyses included sex, age, disease duration and the clinical parameters: age at diagnosis, malar rash, discoid or subacute cutaneous lesions, photosensitivity, oral ulcers, arthritis, serositis, renal, neurological or hematological disorder. The SPSS 12.0 statistical software package (SPSS Inc., Chicago, IL) was used for all calculations.

## Results

### Antimalarial treatment associates with low TNFα serum levels in SLE patients

**Figure 1:**
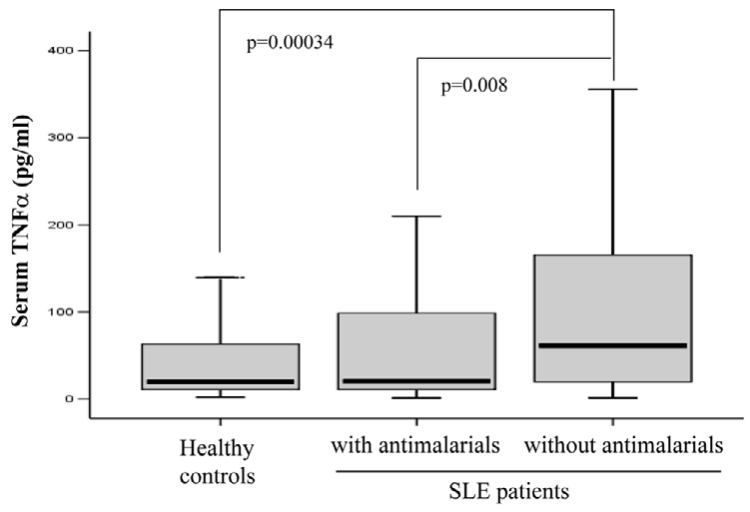
Antimalarial treatment downregulates tumor necrosis factor (TNF)α serum levels in systemic lupus erythematosus (SLE) patients. TNFα serum levels were quantified by ELISA techniques in 171 SLE patients and 215 healthy controls. All patients were classified as users or nonusers of antimalarial drugs during the last three months before sampling. Differences were evaluated by the Mann-Whitney *U *test.

Quantification of TNFα levels in the serum of 171 SLE patients and 215 healthy controls (Table 2) showed a significantly higher amount of this cytokine in the entire patient population compared with controls (*p *= 0.020, Mann-Whitney *U *test). Spearman's rank correlation test did not show any significant relationship between treatment with corticosteroids or immunosuppressive drugs or the clinical features age at diagnosis, malar rash, discoid or subacute cutaneous lesions, photosensitivity, oral ulcers, arthritis, serositis, or renal, neurological or hematological disorder and TNFα serum levels. However, a highly significant negative correlation was detected between the use of antimalarial drugs during at least three months before sampling and the concentration of serum TNFα (ρ = -0.296, *p *= 0.008, Spearman's test). In fact, when patients were stratified according to treatment (Table 2), no differences were detected between controls and patients under antimalarial treatment, either alone or combined with corticosteroids, whereas increased levels were observed in untreated patients and those with corticosteroid or other immunosuppressive therapies. Kruskal-Wallis test analysis did not show significant differences among treatments, probably due to the reduced number of patients in each group after stratification. Thus, when patients were classified as users or nonusers of antimalarial drugs (Figure 1), we found that patients without antimalarial treatment had significantly higher levels of serum TNFα (median value, 61.45) than both healthy controls (19.66, *p *= 0.00034) and SLE patients receiving this drug (20.60, *p *= 0.008). These results suggest that antimalarial treated SLE patients do not have the increased TNFα production usually found in lupus patients, showing serum levels similar to healthy controls.

### Antimalarial-mediated inhibition of TNFα is associated with TNFα and IL-10 promoter genotypes

It has been previously shown that the TNFα genotype at the -308 position (A/G) regulates basal TNFα mRNA levels, although no significant differences were detected in healthy controls at the protein level [10]. To ascertain the possible influence of this functional SNP on the association between antimalarial treatment and decreased TNFα levels, patients and controls were genotyped and classified as genetically high (-308AA or AG) or low (-308GG) TNFα producers and TNFα serum levels were evaluated in patients who used antimalarial drugs and those who did not (Table 3). Highly significant differences between the two groups were detected among high TNFα producer patients (*p *= 0.001), whereas no differences between users and nonusers were observed among genetically low TNFα producers.

**Figure 2:**
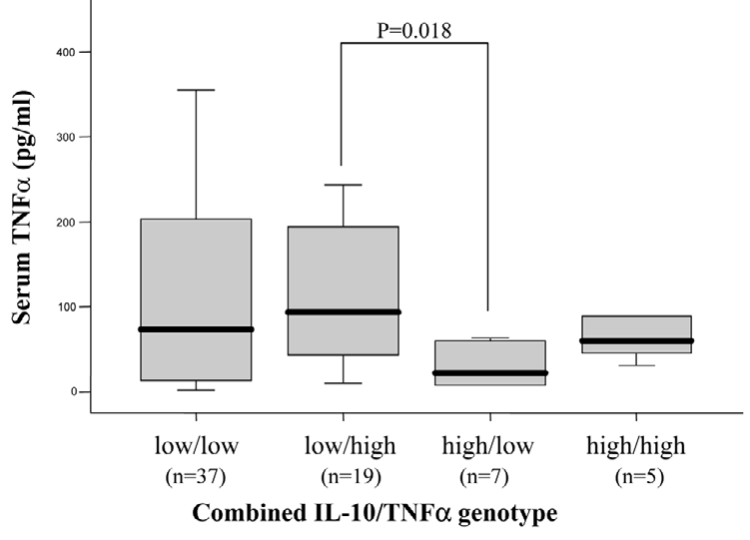
Combined IL-10/tumor necrosis factor (TNF)α genotype influences TNFα serum levels in SLE patients without antimalarial treatment. After determination of the allele present at positions -1,082 and -308 of the IL-10 and TNFα gene promoters, respectively, patients were classified as genetically high (GG) or low (AA/AG) IL-10 producers and high (AA/AG) or low (GG) TNFα producers. Box plots represent TNFα serum levels in SLE patients not receiving antimalarial therapy classified according to the four possible IL-10/TNFα combined genotypes. Differences were evaluated by the Mann-Whitney *U *test.

It is known that the two cytokines TNFα and IL-10 are mutually regulated and that, similar to TNFα, IL-10 levels are genetically determined. We thus wished to evaluate the possible role of functional IL-10 genotypes on the suggested antimalarial-mediated TNFα downregulation. All individuals were accordingly classified as high (GG) and low (AA/AG) genetic IL-10 producers by determination of the allele present at the -1,082 position. Table 4 indicates that IL-10 genotype is able to influence TNFα serum levels in SLE patients, as significant differences between users and nonusers of antimalarial treatment were detected among low IL-10 producers (*p *= 0.005). No significant variations were observed among high IL-10 producers. In conjunction, these results suggest a relationship between antimalarial treatment and low TNFα serum levels in genetically high TNFα and low IL-10 producing SLE patients. This association was probably due to the high TNFα levels of this group. In fact, when patients were classified in the four possible combined IL-10/TNFα genotypes, we found that TNFα serum levels in SLE patients without antimalarial treatment were influenced by both cytokine polymorphisms (Figure 2). Low IL-10/high TNFα patients presented significantly higher levels than high IL-10/low TNFα producers (93.19 versus 22.19, *p *= 0.018) whereas patients with balanced cytokine production (low/low and high/high) presented intermediate values (73.61 and 59.97, respectively).

### Combined IL-10 and TNFα genotype influences response to antimalarial treatment in SLE patients

Finally, in order to examine the role of IL-10 and TNFα genetic polymorphisms as predictors of response to treatment, we selected those patients who had been users of antimalarial agents for more than three years without the need for any other specific SLE therapy, thus indicating a successful response to treatment. Among the 192 patients previously genotyped for SNPs at both cytokine genes, we found that 40 patients (20.83%) were good responders to antimalarial therapy whereas another 74 (38.54%) were also users of this treatment but required the combination with corticosteroids or immunosuppressive drugs. Table 5 shows an overrepresentation of the high TNFα genotype in the good responder group. Therefore, using logistic regression modeling, we evaluated the influence of single and combined IL-10 and TNFα functional genotypes on the response to antimalarial therapy. Table 6 shows a significant association between carriage of the high TNFα producer genotype and good response to antimalarial drugs (OR 2.25, 95%CI 1.11–4.58, *p *= 0.024), whereas the IL-10 genotype did not show any significant association. However, when combined genotypes were analyzed, only the low IL-10/high TNFα genotype was significantly associated (OR 3.13, 95%CI 1.41–6.92, *p *= 0.005). Analysis of clinical features indicated that the group of 40 patients who were long-term users of antimalarial drugs without requiring any other specific treatment was characterized by lower frequency of serositis and nephritis when compared with the remainder of the patients (*p *= 0.014 and 0.003, respectively). No significant differences were detected with respect to patient age (46.25 ± 12.95 years versus 47.50 ± 15.07) or disease duration (12.68 ± 7.75 years versus 13.28 ± 7.86). Moreover, previous associations were sustained in the multivariate analysis after adjusting for sex, age, disease duration and clinical parameters. Therefore, these results indicate that, in addition to the TNF2 allele, carriage of the low IL-10 producer genotype is required to become a very good responder patient to antimalarial treatment.

## Discussion

In this study we show that SLE patients receiving single or combined treatment with antimalarial drugs have TNFα serum levels similar to healthy controls, whereas untreated patients and those receiving corticosteroid or immunosuppressive therapies presented increased amounts of this cytokine. These results suggest a very valuable effect of antimalarial treatment by means of the downregulation of *in vivo *TNFα levels. Although these pharmacological agents have been widely used as disease-modifying antirheumatic drugs mainly in the treatment of SLE and rheumatoid arthritis [1], their mechanisms of anti-inflammatory actions have not yet been completely understood. Several *in vitro *experiments have demonstrated that quinacrine and related drugs decreased the release of pro-inflammatory cytokines induced by LPS in macrophages [4-7,15]. In mouse models, it has been reported that chloroquine may protect mice from lethal challenge by CpG oligonucleotides and LPS and may decrease serum TNFα and IL-6 in rats injected with sublethal doses of both stimuli [16]. To the best of our knowledge, however, this is the first report demonstrating an *in vivo *association between the use of antimalarial therapy and low levels of serum TNFα, suggesting that the disease-modifying antirheumatic effect of these drugs may be mediated, at least in part, by a strong downregulatory effect on TNFα production.

However, association between antimalarial treatment and TNFα serum levels seems to be influenced by polymorphisms of the genes encoding TNFα and IL-10, indicating that this advantageous connection may only be completely valuable for patients with a specific genotype. Constitutive and induced TNFα and IL-10 production have important interindividual variations that are genetically regulated by SNP at their promoters [8-12]. Our data indicate that antimalarial therapy plays a role in the TNFα production of patients who are genetically high TNFα producers. These patients probably have the highest TNFα transcription rates [9] and consequently the highest serum levels. Although the mechanisms of *in vitro *antimalarial-mediated TNFα inhibition [4-7] are not yet entirely known, it has been reported that chloroquine inhibited LPS-induced TNFα transcription [6,7], interfering with mitogen-activated protein kinase signaling [17]. Thus, our results suggest that antimalarial agents require a high rate of TNFα transcription to achieve the maximal inhibitory effect. On the other hand, though the relevance of IL-10 was already known in lupus disease, the influence of genetic polymorphisms at the IL-10 promoter on treatment outcome after the use of antimalarials was surprising. Results indicated an involvement of antimalarial treatment in the amount of serum TNFα in SLE patients with a low IL-10 genotype. The limited TNFα downregulatory effect observed in genetically high IL-10 producing patients might be explained by the regulatory feedback mechanism that controls the production of both cytokines, which would lead to a decrease in the TNFα transcription rate in patients producing elevated amounts of IL-10. Supporting this, it has been reported that high IL-10 levels were associated with less effective clearance of *Plasmodium falciparum *parasites in patients receiving antimalarial therapy [18].

Our data support the idea that the actions of cytokines are profoundly conditioned by the presence of other cytokines, particularly in the case of IL-10 and TNFα, which have opposing roles in systemic inflammatory responses. Thus, on the basis of our previous results, we evaluated the role of the interaction between IL-10 and TNFα genotypes in regulating the response to antimalarial treatment in SLE patients. A strong association was found between carriage of the combined genotype low IL-10/high TNFα and the use of antimalarials for more than three years without the need for any other specific SLE treatment (good responder patients), although the single analysis of the IL-10 genotype did not show significant results. Moreover, the relationship between this combined genotype and treatment outcome was higher than that obtained after single analysis of the gene encoding TNFα, since high IL-10/high TNFα producers were not overrepresented among good responder patients. Taken in conjunction, these results thus indicate that determination of TNFα and IL-10 alleles at the onset of the disease may help identify more suitable candidates for antimalarial treatment and could be used as a genetic predictor of clinical outcome. We would expect SLE patients who are carriers of the pro-inflammatory genotype low IL-10/high TNFα to develop a mild disease presenting a good course under antimalarial therapy. Most of these patients probably developed SLE due to the effect of environmental factors added to their genetically determined high TNFα levels, which could not be modulated by the low production of IL-10. TNFα is a pro-inflammatory cytokine that has been found at elevated levels in the serum of patients suffering SLE and other autoimmune diseases [19,20]; it has also been suggested that TNFα genotype influences their susceptibility [10,21,22] and, possibly, their clinical response to treatment. In these patients, the elevated TNFα levels may be involved in diverse pathological mechanisms and, therefore, a clinical benefit is to be expected under a treatment that diminishes TNFα production.

Under the assumption that the elevated TNFα levels found in patients with various chronic inflammatory diseases are deleterious, several anti-TNFα therapies are now available to block the action of TNFα. Actually, TNFα-blockage with antibodies has been of unquestionable clinical benefit to many patients with rheumatoid arthritis, Crohn's disease, psoriasis and, more recently, ankylosing spondilitis [20,23-26]. However, it has been reported that response to this treatment is also influenced by genetic polymorphisms at FcγRIII [27], HLA-DRB1 [28], lymphotoxin-α [29] and TNFα alone [30] or combined with IL-10 [31] or lymphotoxin-α [28]. In fact, TNFα antagonists seem to be more effective in genetically low TNFα producer patients [30] or with the combined high IL-10/low TNFα genotype [31]. Several authors have also proposed the use of TNFα lowering agents in the treatment of lupus disease [20,32,33]. Supporting this, an open label study of infliximab in six patients with SLE indicated that TNFα blockage might have a therapeutically beneficial effect, although autoantibodies to double-strained DNA and cardiolipin were increased [34]. This trend toward an augmented autoantibody production frequently accompanying this treatment was not reported after the use of antimalarials. Given the therapeutical relevance of these results, new studies need to be designed to evaluate the use of these drugs as TNFα downregulators in the treatment of genetically low IL-10/high TNFα producer patients with SLE and other inflammatory diseases who, in addition, are poor responders to TNFα blockage with antibodies.

The limitations of the work were the lack of a prospective longitudinal study analyzing TNFα levels before and after treatment with antimalarials and other SLE therapies and the absence of standardized validated measures of SLE activity.

## Conclusion

Our results demonstrate a relationship between antimalarial treatment and low TNFα serum levels in SLE patients that were influenced by polymorphisms at the IL-10 and TNFα gene promoters. Therefore, our findings may have an important clinical application through the identification of patients who are the most likely to benefit from antimalarial therapy.

## Abbreviations

CI = confidence interval; ELISA = enzyme-linked immunosorbent assay; IL = interleukin; LPS = lipopolysaccharide; NSAIDS = nonsteroidal anti-inflammatory drugs; OR = odds ratio; PCR = polymerase chain reaction; SLE = systemic lupus erythematosus; SNP = single nucleotide polymorphism; TNF = tumor necrosis factor.

## Competing interests

The authors declare that they have no competing interests.

## Authors' contributions

PL performed genetic and immunological assays, data collection and statistical analyses and participated in interpretation of data. JG carried out a detailed review of clinical histories and participated in data collection. LM performed patient selection and participated in data collection. CG participated in the study design, interpretation of data and helped to draft the manuscript. AS conceived the study, participated in its design and coordination, performed genetic assays and statistical analyses and drafted the manuscript.

## Figures and Tables

**Table 1:** Systemic lupus erythematosus patient characteristics and disease parameters

Total SLE patients	192
Sex (female/male)	178/14
Age (mean ± sd)	47.24 ± 14.63
Age at diagnosis (mean ± sd)	33.88 ± 14.53
Disease duration (mean ± sd)	13.16 ± 7.83
	
Clinical manifestations, n (%)	
Malar rash	102 (53.1)
Discoid lesions	38 (19.8)
Subacute cutaneous lesions	33 (17.2)
Photosensitivity	99 (51.6)
Oral ulcers	63 (32.8)
Arthritis	139 (72.4)
Serositis	48 (25.0)
Renal disorder	56 (29.2)
Neurological disorder	15 (7.8)
Hematological disorder	111 (57.8)
	
Treatment, n (%)	
None/NSAIDS	23 (12.0)
Antimalarials	114 (59.4)
Corticosteroids	126 (65.6)
Immunosuppressive drugs^a^	41 (21.4)

^a^Methotrexate, azathioprine, cyclophosphamide, cyclosporine A or mycophenolate mophetil. NSAIDS, nonsteroidal anti-inflammatory drugs; SD, standard deviation; SLE, systemic lupus erythematosus.

**Table 2:** Tumor necrosis factor-α serum levels in healthy controls and systemic lupus erythematosus patients

	*n*	TNFα (pg/ml)^a^	*p*
Healthy controls	215	19.66 (53.93)	0.020
SLE patients	171	33.57 (132.55)	
Patient treatment:			
None/NSAIDS	21	60.78 (158.15)	0.136
Antimalarials	36	16.64 (165.57)	
Antimalarials and corticosteroids	54	24.95 (88.49)	
Corticosteroids	26	60.01 (145.67)	
Immunosuppressive drugs^b ^alone or with corticosteroids	22	105.34 (195.29)	
Antimalarials, corticosteroids and immunosuppressive drugs^b^	12	16.89 (50.70)	

^a^TNFα values are median (interquartile range). ^b^Methotrexate, azathioprine, cyclophosphamide, cyclosporine A or mycophenolate mophetil. Differences were evaluated by Mann-Whitney or Kruskal-Wallis nonparametric testing. NSAIDS, nonsteroidal anti-inflammatory drugs; SLE, systemic lupus erythematosus; TNF, tumor necrosis factor.

**Table 3:** TNFα levels in high and low TNFα producing patient users and nonusers of antimalarial treatment

TNFα genotype	Healthy controls		Patients without antimalarial drugs		Patients with antimalarial drugs		*p*
	*n*	TNFα (pg/ml)^a^	*n*	TNFα (pg/ml)^a^	*n*	TNFα (pg/ml)^a^	

High (AA/AG)	53	20.06 (125.63)	24	83.55 (171.64)	48	16.88 (54.89)	0.001
Low (GG)	162	18.83 (40.67)	45	58.56 (168.94)	54	26.00 (154.67)	0.516

^a^Values are median (interquartile range). Differences between users and nonusers of antimalarial therapy were evaluated by the Mann-Whitney U test. TNF, tumor necrosis factor.

**Table 4:** TNFα levels in high and low IL-10 producing patient users and nonusers of antimalarial treatment

IL-10 genotype	Healthy controls		Patients without antimalarial drugs		Patients with antimalarial drugs		*p*
	*n*	TNFα (pg/ml)^a^	*n*	TNFα (pg/ml)^a^	*n*	TNFα (pg/ml)^a^	

Low (AA/AG)	179	18.55 (49.27)	56	80.88 (201.48)	79	20.94 (84.97)	0.005
High (GG)	36	21.74 (58.42)	12	51.91 (51.75)	23	20.03 (134.44)	0.797

^a^Values are median (interquartile range). Differences between users and nonusers of antimalarial therapy were evaluated by the Mann-Whitney *U *test. TNF, tumor necrosis factor.

**Table 5:** IL-10 and TNFα genotypes of systemic lupus erythematosus patients stratified by treatment

Treatment	TNFα genotype		IL-10 genotype	
	High (AA/AG)	Low (GG)	High (GG)	Low (AA/AG)

None/NSAIDS	6 (26.1)	17 (73.9)	4 (17.4)	19 (82.6)
Antimalarials	23 (57.5)	17 (42.5)	6 (15.0)	34 (85.0)
Corticosteroids	8 (28.6)	20 (71.4)	6 (22.2)	21 (77.8)
Antimalarials and corticosteroids	25 (41.7)	35 (58.3)	16 (26.7)	44 (73.3)
Immunosuppressive drugs^a ^alone or with corticosteroids	13 (48.1)	14 (51.9)	3 (11.1)	24 (88.9)
Antimalarials, corticosteroids and immunosuppressive drugs^a^	5 (35.7)	9 (64.3)	3 (21.4)	11 (78.6)

Values are n (%). ^a^Methotrexate, azathioprine, cyclophosphamide, cyclosporine A or mycophenolate mophetil. NSAIDS, nonsteroidal anti-inflammatory drugs; TNF, tumor necrosis factor.

**Table 6:** Association between IL-10 and TNFα genotypes and response to antimalarial treatment

Genotype	Healthy controls n (%)	SLE patients		Univariate analysis		Multivariate analysis^a^	
		Good responders to antimalarials n (%)	Others n (%)	OR (95% CI)	*p*	OR (95% CI)	*p*

-1,082 IL-10							
Low (AA/AG)	292 (85.1)	34 (85.0)	119 (78.8)	Referent		Referent	
High (GG)	51 (14.9)	6 (15.0)	32 (21.2)	0.66 (0.25–1.70)	0.386	0.59 (0.20–1.78)	0.354
							
-308 TNFα							
Low (GG)	265 (77.3)	17 (42.5)	95 (62.5)	Referent		Referent	
High (AA/GA)	78 (22.7)	23 (57.5)	57 (37.5)	2.25 (1.11–4.58)	0.024	2.67 (1.20–5.97)	0.016
							
Combined IL-10/TNFα							
Low/Low	224 (65.3)	12 (30.0)	75 (49.7)	Referent		Referent	
Low/High	68 (19.8)	22 (55.0)	44 (29.1)	3.13 (1.41–6.92)	0.005	4.20 (1.66–10.63)	0.002
High/Low	41 (12.0)	5 (12.5)	19 (12.6)	1.64 (0.52–5.24)	0.400	1.77 (0.47–6.63)	0.397
High/High	10 (2.9)	1 (2.5)	13 (8.6)	0.48 (0.06–4.02)	0.499	0.43 (0.04–4.21)	0.472
Trend test				0.021		0.011	

Association was calculated by unconditional logistic regression modeling using good response to antimalarial treatment as the dependent variable. ^a^Adjusted for sex, age, disease duration and clinical parameters: malar rash, discoid lesions, subacute cutaneous lesions, photosensitivity, oral ulcers, arthritis, serositis, renal disorder, neurological disorder, hematological disorder and age at systemic lupus erythematosus (SLE) diagnosis (continuously). CI, confidence interval; OR, odds ratio; TNF, tumor necrosis factor.
